# Lac Repressor Mediated DNA Looping: Monte Carlo Simulation of Constrained DNA Molecules Complemented with Current Experimental Results

**DOI:** 10.1371/journal.pone.0092475

**Published:** 2014-05-06

**Authors:** Yoav Y. Biton, Sandip Kumar, David Dunlap, David Swigon

**Affiliations:** 1 Department of Mathematics, University of Pittsburgh, Pittsburgh, Pennsylvania, United States of America; 2 Department of Cell Biology, Emory University School of Medicine, Atlanta, Georgia, United States of America; Hungarian Academy of Sciences, Hungary

## Abstract

Tethered particle motion (TPM) experiments can be used to detect time-resolved loop formation in a single DNA molecule by measuring changes in the length of a DNA tether. Interpretation of such experiments is greatly aided by computer simulations of DNA looping which allow one to analyze the structure of the looped DNA and estimate DNA-protein binding constants specific for the loop formation process. We here present a new Monte Carlo scheme for accurate simulation of DNA configurations subject to geometric constraints and apply this method to Lac repressor mediated DNA looping, comparing the simulation results with new experimental data obtained by the TPM technique. Our simulations, taking into account the details of attachment of DNA ends and fluctuations of the looped subsegment of the DNA, reveal the origin of the double-peaked distribution of RMS values observed by TPM experiments by showing that the average RMS value for anti-parallel loop types is smaller than that of parallel loop types. The simulations also reveal that the looping probabilities for the anti-parallel loop types are significantly higher than those of the parallel loop types, even for loops of length 600 and 900 base pairs, and that the correct proportion between the heights of the peaks in the distribution can only be attained when loops with flexible Lac repressor conformation are taken into account. Comparison of the *in silico* and *in vitro* results yields estimates for the dissociation constants characterizing the binding affinity between O1 and Oid DNA operators and the dimeric arms of the Lac repressor.

## Introduction

In living organisms, the level of protein production must be optimally adjusted in order to ensure the adaptation of the organisms to environmental changes. Therefore, the mechanism of transcription regulation must be tightly controlled by elements such as DNA binding proteins that, often with the help of an inducer molecule, bind to specific DNA sites and promote or repress the transcription activity of RNA polymerase. The action of some of these regulatory proteins involves DNA looping, a process in which the protein binds simultaneously to two sequentially remote sites along a DNA molecule and brings these sites to a close proximity by bending the intermediate DNA segment [1], [2].

The functional relevance of DNA looping in transcription regulation has been revealed by the intensively studied, and so far the best understood, regulatory mechanism of the *lac* operon in *E. coli* [3]. In this system one dimeric arm of the Lac repressor tetramer binds to an operator site (O1) near the promoter resulting in inhibition of expression of the genes coding the proteins involved in the metabolism and transport of lactose. A loop can be formed by a simultaneous binding of the other dimeric arm of the Lac repressor tetramer to one of two auxiliary operators located 410 bp (O2) downstream and 82 bp (O3) upstream of O1. Such a looped state has a greater transcriptional repression due to a stabilization of the interaction between the Lac repressor and the operator O1 [4], which, in part, can be explained by the statistical mechanics of the system: the fact that the looping protein is bound to one operator increases its concentration in the vicinity of the other operator. The process has been studied extensively *in vitro* by gene expression measurements [1], [2], [5].

Various techniques have also been developed to study DNA looping *in vivo*. Since the formation of a DNA loop in a linear DNA segment results in a shortening of the mean end-to-end distance of DNA ends, it can be detected and quantified by single-molecule manipulation experiments. In one such experiment, called tethered particle motion (TPM) experiment, a single linear DNA molecule with two protein binding sites is attached at one of its two ends to a microscope coverslip and at the other to a large bead that can be tracked by light microscopy, and then looping protein is inserted into the solution. By observing the position of the bead as a function of time one can estimate the length of the DNA tether; a shortening of the tether that persists over a predetermined time window is interpreted as an indicator of the presence of a loop. By measuring the mean time spent in looped and unlooped states one can track the kinetics of loop formation and breakage [6]. This type of setup was first used for measurements of the relative motion between a single DNA and RNA polymerase during transcription [7], [8], and later was utilized for the study of lac-repressor mediated loop formation [6].

Because of the symmetry of the two identical dimeric arms of the Lac repressor, an operator can bind to each arm in two possible orientations yielding four distinct loop types: two parallel loop types P1 and P2, and the two antiparallel types A1 and A2. Similar loop types were suggested to be formed by the binding of two dimeric galR proteins to two operators in the *E. coli gal* operon [9]. In addition, arguments have been presented for the existence of another loop type in which Lac repressor attains an extended conformation in which the hinge, i.e., the four-helix bundle tetramerization domain, permits the two dimeric arms of the Lac repressor to extend out from the nearly rigid “V” conformation [10]. The evidence for existence of an extended Lac repressor conformation ranges from electron microscopy [11], modeling of DNA footprinting and looping free energy measurements [12], gene expression by very small Lac repressor-DNA loops [13], modeling of *in vitro* binding assays [14], and FRET measurements of distances between DNA constructs bound to LacI [15], [16]. Especially the latest FRET results of Haeusler et al. [17] show convincingly that the flexibility of Lac repressor during looping process cannot be ignored.

The TPM measurements can be used to shed light on the structure of the loop. If the length of the loop is known from the positions of binding sites for the looping protein, then the change in end-to-end distance measured by TPM can be used to infer geometrical and deformational details of the looped protein-DNA structure. The influence of the flexibility of the binding protein on loop formation was investigated by Vanzi et al. [18], who showed an increased mean duration of looped states with a more flexible Lac repressor hinge region. Recent TPM experiments showed that the motion of the tethered bead gives rise to two-peak distribution [19] of the values of the end-to-end distance that correspond to looped states. Unfortunately, TPM measurements do not provide direct information about the structure of the loop, and in order to confirm or disprove any hypotheses about the type of the observed loop one needs to construct a model that provides, for each loop type, the end-to-end distribution of DNA and hence the observable length of the tether.

A number of coarse-grained DNA models can serve as a framework for the study of the statistical mechanics of DNA looping [20]. Continuum elastic rod model, which treats DNA as an ideal elastic rod, i.e., thin elastic body that is inextensible, intrinsically straight, transversely isotropic and homogeneous [21] is the basis of the classical helical worm-like chain model [22] that has been used in many papers to study DNA supercoiling, topoisomer distributions, and single-molecule stretching experiments. More recently discrete base-pair level models have been developed, which account for the dependence of DNA elasticity on sequence [23], [24]. Statistical mechanics of DNA molecules of length in the order of a few hundreds to several thousands base pairs has been studied using Gaussian sampling approach [24], [25], [26], [27], which utilizes the multivariate quadratic form of DNA deformational energy. The Gaussian sampling method can generate efficiently a very large canonical ensemble (up to 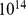 configurations) of uncorrelated configurations. A model of this type has been used in the interpretation of TPM looping experiments reported in [19], [27] to suggest that the observed end-to-end distribution is the result of the competition of several loop types (extended Lac repressor loop was not among them), each of which corresponds to particular geometrical constraints applied to the DNA by the protein.

However, a significant drawback of Gaussian sampling is that only a very small fraction of sampled configurations obeys prescribed end conditions. Furthermore, this method cannot be utilized when other contribution to the total energy, such as the intra-molecular electrostatic energy of a DNA molecule in solution, is taken into account [28]. For these reasons Metropolis Monte Carlo simulations [29], [30], [31] have been developed, based on the classical Markov Chain Monte Carlo method of Metropolis and Hastings [32], in which a model DNA is changed by small deformations and any new configuration is accepted with a probability that depends on the difference between its energy and the energy of the previous configuration.

In this paper we describe a new Metropolis Monte Carlo algorithm which enables us to generate large canonical ensembles of DNA configurations subject to any composition of geometric and topological constraints, including looped configurations of given topology, closed configurations, and configurations with preassigned end-to-end (or, more generally, site-to-site) distance. This algorithm utilizes explicit expression for the Jacobian of the mapping between DNA deformational parameters and end-to-end conditions, derived in [33], and successfully overcomes a significant difficulty: applying reversible perturbations that preserve the given geometric constraints. This scheme differs from other Monte Carlo schemes utilizing local perturbations such as the crankshaft move and global perturbations such as the slithering move, introduced in [29], which cannot be used for a non-homogenous sequences and are limited for circularized molecules or molecules subject to fully clamped end conditions.

We also present the results of the application of the new Monte Carlo scheme to simulations of TPM studies of Lac repressor mediated DNA looping. In these simulations, as in the experiment, DNA is attached to a rigid substrate at one end and fluctuating bead at the other, and a loop may be formed by bridging operator sites with bound Lac repressor. The scheme enables us to take into account the fluctuations of the looped segment of the DNA, an effect that was neglected in [19], [27]. We calculate looping probabilities for a prescribed loop type and use this information to analyze the likelihood that a loop of a particular length has a given topology. By comparing our computational results to our TPM experimental results we estimated also the dissociation constants associated with the binding of the Lac repressor to each of the operators.

## Methods

Our theoretical model is based on the familiar naturally discrete model for DNA elasticity [23], [34], [28] in which the intramolecular electrostatic interactions and their dependence on the ionic strength in the aqueous media are taken into account [28], [33]. In our simulations of TPM experiments all the excluded volume effects such as the impenetrability of the DNA molecule, the bead, and the plate, are taken into account. We start with an introduction of the underlying theory in the following subsection.

### DNA model

**Figure 1:**
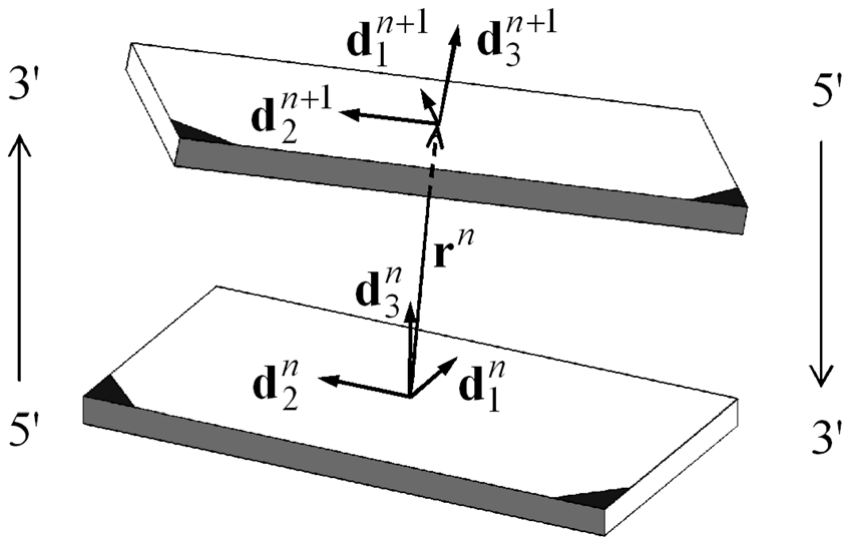
Base-pair step. A schematic drawing of the two adjacent base pairs forming the 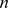-th step of DNA. Each nucleotide base in the 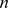-th base pair is covalently bonded at its darkened corner to one of the two sugar phosphate backbone chains. The direction of that oriented chain is indicated by a light-face arrow; the chain itself is not shown. The gray-shaded long edges are in the minor groove of the DNA.

We here employ the theory presented in [23], [28], [33], in which the energy of a DNA molecule with 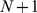 base pairs is determined when there is given, for each 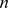, both the location 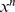 of the barycenter of the 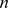-th base pair and an orthonormal triad 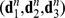 that is embedded in the base pair as shown in Figure 1. The total energy, 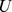, of a DNA configuration is taken to be the sum of elastic energy 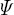 and electrostatic energy 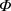,
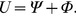
(1)


**Figure 2:**
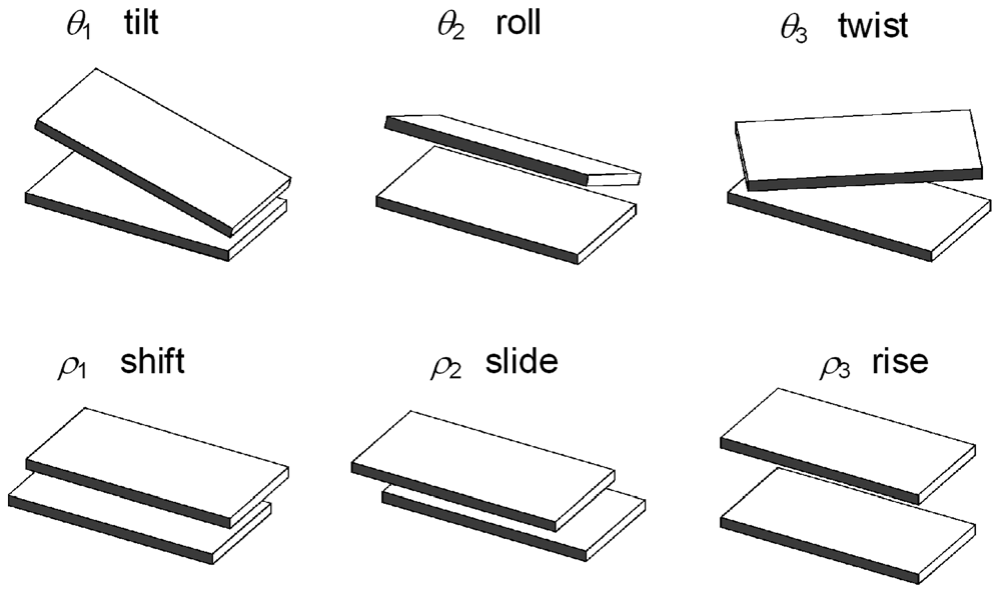
Kinematical variables. Schematic representations of the kinematical variables that describe the relative orientation and displacement of consecutive base pairs. Each drawing illustrates a case in which one of the kinematical variables has a positive value and the others (with the exception of 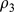) are set equal to zero.

The elastic energy 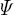 of a configuration is taken to be the sum over 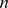 of the energy 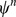 of interaction between the 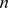-th and the 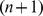-th base pairs, i.e.,
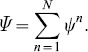
(2)The local elastic energy 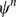 associated with the 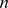-th base-pair step is given by a function of the relative position and orientation of the 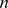-th and 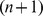-th base pairs, which can be parameterized by six kinematical variables: shift, slide, and rise, (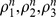), which describe local shearing and extension (i.e., stretching), and tilt, roll, and twist, (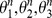), which describe local bending and twisting of the molecule [35], [28] (See Figure 2). The displacement deformations (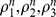) have only a small influence on the end-to-end distribution of long DNA compared with the angular deformations, because each angular deformation contributes a change in the end position that is proportional to the distance of the end from the location of that deformation. A single displacement deformation contributes, on average, 0.1 nm to the position of the end of the DNA, while an average bending deformation of 5 degrees, if occurring in the middle of a 300 bp segment, contributes about 4.5 nm to the position of the end. Therefore, in order to reduce the number of kinematical variables and make the calculation computationally feasible we shall assume that the values of the shift, slide, and rise of each base-pair step are constants. This simplification reduces by one half the number of degrees of freedom results in about 10 times shorter simulation times.

As a further simplification, the local elastic energy 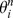 is assumed to be a quadratic form in the excess tilt 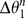, the excess roll 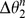, and the excess twist 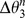 defined as
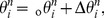
(3)where 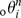 are the intrinsic values appropriate to a stress-free (minimum energy) state of the 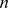-th base-pair step. Thus,
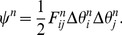
(4)The elastic moduli, 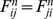, and the intrinsic parameters are constants that may depend on the nucleotide composition of the 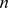-th and (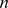
+1)-th base pairs. Here, for simplicity, we assume that the molecules are transversely isotropic and that their elastic properties are independent of the sequence. This is done in order to determine whether the experimental results can be explained without resorting to sequence-dependent effects. Thus, we assume
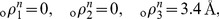
(5)

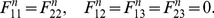
(6)for 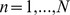. In the present case we take a value for the two bending moduli that gives rise to a persistence length of 476 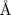, namely,
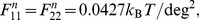
(7)with 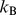 Boltzmann's constant and 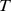 the temperature which is taken to be 300 K.

The electrostatic energy of a configuration has the form
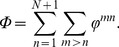
(8)where 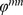 is the electrostatic energy associated with the interaction of the 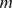-th and the 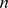-th base pairs of the DNA molecule. As an approximation it is assumed that the two negative charges associated with each base pair are located at the barycenter, 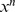, of that base pair [36]. Following Manning's theory of charge condensation [37] the energy 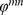 is given by (See also the discussion of Westcott et al. [36]),
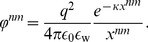
(9)where
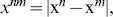
(10)

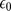
 is the permittivity of free space, and 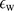 is the dielectric constant of water. In accord with Manning's theory [37], [38], 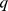 is set equal to 24% of the charge of the two phosphate groups associated with each base pair, i.e., 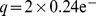, where 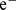 is the charge of an electron. The DNA molecule is assumed to be in a solution of water and monovalent salt (e.g., NaCl) of concentration 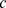. The Debye screening parameter 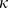 is given by the formula
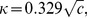
(11)in which 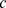 is measured in the units of moles per liter and 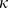 in the units of 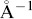. For the present work we take 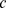 to be equal to its physiological value: 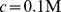. Although the present value of the salt concentration, 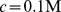, yields a non-negligible repulsive intra-molecular interaction, under our assumptions, the magnitude of the resulted repulsive force between sequentially remote sites that are almost in an immediate contact, is not strong enough to avoid self penetration. We therefore implemented the theoretical model introduced in [33] in which the DNA is regarded as a tube-like structure composed of the union of rigid cylinders connected by spherical joints. Accordingly, every generated configuration with self-penetration is rejected.

### Generation of constrained configurations

When, as in the present work, the displacement parameters are given as preassigned constants, a configuration of a DNA molecule with 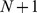 bp is determined (up to a rigid body transformation) by the set of 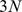 angular variables 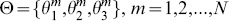. A loop, comprised of 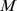 bp, with 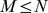, is formed when two sequentially remote sites along a single DNA molecule are attached to a single linker protein and, as a result, are brought into a close proximity. A linker protein that is simultaneously attached to two base pairs, say 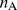 and 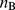, with 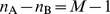, confines the two termini of the intermediate DNA segments to six constraints prescribing the relative orientation and displacement between the bound base pairs:
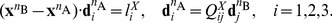
(12)The numbers 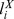 and 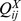 are the components of the displacements and the orthogonal transformation between the two termini of the loop, and are determined solely by the structure of the linker protein. Here, for simplicity, we associate the boundary conditions with the middle base pair of each binding site, i.e., the termini of the loop are taken to be base pairs 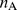 and 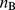. Because a single linker protein may form loops of several distinct topologies, we indicate the topology by superscript 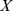. A looped configuration satisfying equations (12) gives rise to independent constraints of the form:
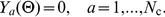
(13)The number of constraints, 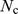, is six, but can be reduced to any value from one to five by relaxing some of the constraints. For example, 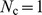, when the loop is free of external moments, i.e., when the two termini of the loop are free to rotate (as in the case of spherical joints), and hence only the distance 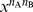 is prescribed, or, 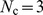, when only the left hand side of equations (12) is applied.

A looped DNA molecule in a TPM experiment can be regarded as a composition of 3 segments separated by the middle base pair in each of the two binding sites: the two terminal segments with base pairs (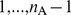) and (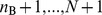), are attached to a planar plate (e.g., a microscope cover-slip) and a bead, respectively, and the intermediate segment that may form a loop.

Metropolis Monte Carlo technique requires that a configuration satisfying constraints (13) is randomly perturbed in such a way that the constraints are still satisfied. Suppose that in a given move the chosen segment is the intermediate looped segment. To perform a change in the configuration of the looped segment that does not perturb equations (13), we randomly select four base-pair steps, 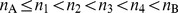, and change only the 12 angular variables, 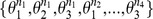 while holding all the remaining variables fixed. For simplicity of exposition we set these variables to be the 12 components of 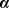,
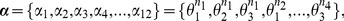
(14)and for the arguments involving a single move in our scheme we regard 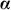 as a configuration. Accordingly, the equations (13) form a system of six nonlinear equations with only 12 unknowns. A linearization of the equations about a looped configuration, 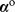, yields
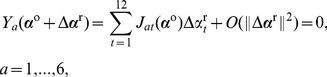
(15)where 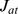 are the entries of the 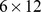 Jacobian matrix, 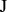, representing the gradient of the functions 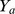 evaluated at 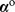. To generate a finite perturbation, 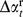, in the components of 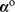, in such a way that the linearized constraints,
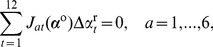
(16)hold, we first calculate the 6 linearly independent 12-dimensional unit vectors 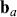 that span 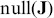, the null space of 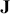. With the basis 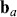, 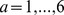, in hand, we set a random move that satisfies the linearized constraints (16):
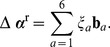
(17)The 6 numbers 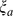 are randomly generated to yield a random vector 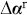 uniformly distributed in the volume bounded by the (projected) hypersphere of radius 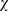, i.e., 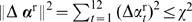.

According to this procedure, whenever 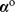 satisfies equations (13) any non-zero variation 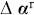 gives a new configuration, 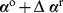 that, although obeys the equations (16), does not necessarily satisfy (with high enough precision) the constraints (13). To complete a single move in our Metropolis algorithm, we calculate the unique correction 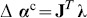 in the subspace, 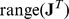, for which 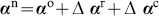 satisfies the constraints (13). Since the correction, 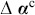, is restricted to a six-dimensional space, the problem is now reduced to a nonlinear system of the six equations (13) with the six unknown components of 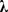. Implicit function theorem and the smoothness of the hyper-surface characterized by equations (13) assure the uniqueness of 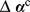 for a small enough value of 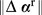. Thus, for the range of values of 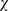 appropriate for the Metropolis Monte Carlo algorithm used here there exists a 1-1 mapping from the configuration 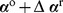 to 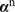. In the calculations performed here we took 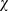 to be equal or less than 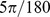. This restricts a single change in each angular variable to be of a magnitude smaller than 5 degrees. The unique solution for 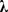 is here calculated using a modified Newton-Raphson scheme which gives a solution, 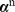, of the equations (13) to within machine accuracy in no more than 3 iterations.

**Figure 3:**
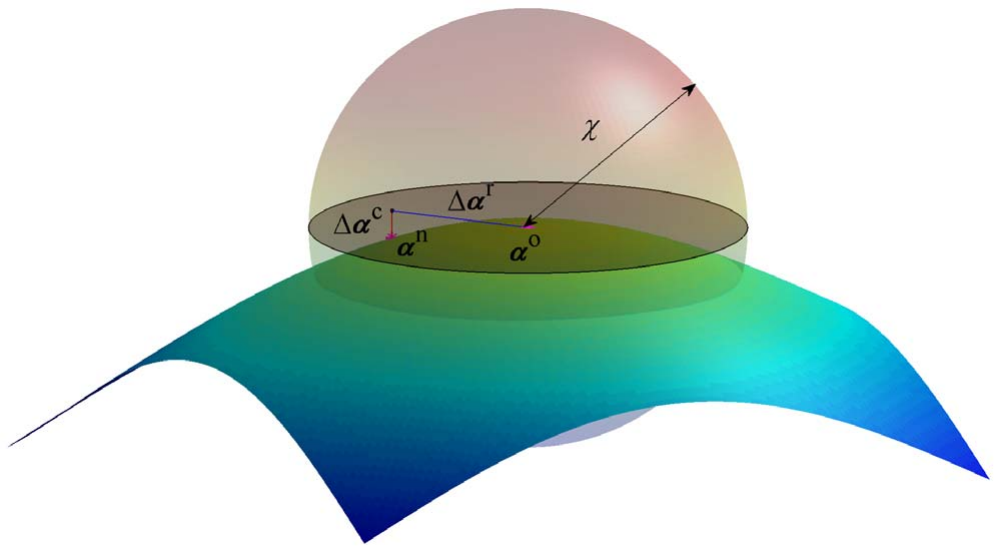
A simple example of the numerical scheme in 3D. A 3D schematic example of the numerical scheme for generating constrained configurations for the Metropolis Monte Carlo computations. The surface can be thought of as a representation of a single constraint for a system with three degrees of freedom. The random move, 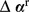 is along the tangent plane to surface (the shaded discoid) spanned by 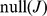, and the correction move, 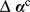 is along the normal to that plane. The complete move must be bound by the shaded sphere, so that the probability to move from 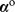 to 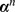 is equal to that of the reverse move.

The method described above can be easily utilized for any number of constraints. For the sake of assuring a strict detailed balance condition, a new configuration 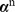 is accepted only if the total move is within the hyper-sphere of radius 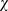 centered at 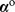, i.e., we require that
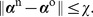
(18)This, together with the uniform distribution of 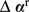 implies that the probability 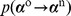 to move from 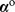 to 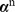 equals 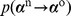. A schematic description of the scheme for 3-dimensional problem with two constraints is shown in Figure 3. The shaded disc represents the null space of 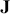, and the normal to the disc describes its orthogonal complement 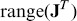. We note that the very important calculation of the entries of the Jacobian matrix 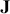 is done based on analytical expressions in a way similar to the numerical scheme for finding equilibrium configurations of DNA molecules introduced in [28]. This improves significantly both the numerical stability and efficiency of the scheme.

### Statistical Mechanics of DNA configurations

To calculate a canonical ensemble of configurations obeying the Boltzmann distribution we utilize Metropolis Monte Carlo algorithm in which configuration is changed incrementally using a move that is acceptable, i.e., in accord with the constraints (13), under strict detailed balance and ergodicity conditions [39], [40] assuring unbiased sampling and possible explorations of the complete configurational space. Then we calculate the total energy of the new configuration and apply the acceptance criteria of the Metropolis scheme. The DNA, the attachment plate, and the bead are all modeled as rigid impenetrable objects and hence any configuration with DNA-DNA, bead-DNA, bead-plate, or DNA-plate spatial overlap are rejected. The impenetrability of DNA is treated in accord with the model introduced in [33], in which a DNA molecule is treated as a union of rigid spheres separated by rigid cylinders.

The standard acceptance criteria according to the Metropolis Monte Carlo scheme [32] used here yield a canonical distribution of configurations. Thus, when the displacement parameters are held fixed, the statistical weight of a configuration 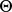 with total energy 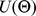 is proportional to the Boltzmann factor 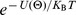. The configurational integral of a molecule has the form
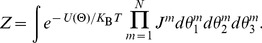
(19)where the integral is taken over the domain of configurations obeying the impenetrability constraints and any other set of geometric constraints relevant to the investigated case. The Jacobian factors 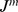 (not to be confused with the Jacobian matrix 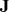 used in the previous subsection) are necessary for the change of variables from canonical parametrization to the non-canonical parametrization used here [41]. This is important because any set of values for the angular variables, 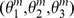, that is rendered from a random number generator with uniform distribution does not yield a sample of the triads (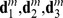) that is uniformly distributed in the group of proper rotations. It can be shown (see e.g., the appendix of [28]) that for the El Hassan and Calladine parametrization used here we have
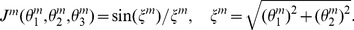
(20)
Equation (20) can also be obtained using the formulation suggested in [41]. The Jacobian 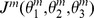 was taken into account in our numerical scheme.

It is convenient, for calculations of loop probability, to use the axis-angle representation of proper rotations, in which a rotation is characterized by a unit vector describing the axis of rotation and a rotation angle about it. The use of this representation is discussed later in this section. The axis of rotation is defined by an azimuthal angle 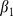 and a polar angle 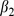, and the rotation about it is given by 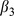. In a similar way to the derivation of equation (20), it can be shown that the Jacobian associated with this parametrization is given by,
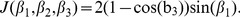
(21)


### Looping probabilities

**Figure 4:**
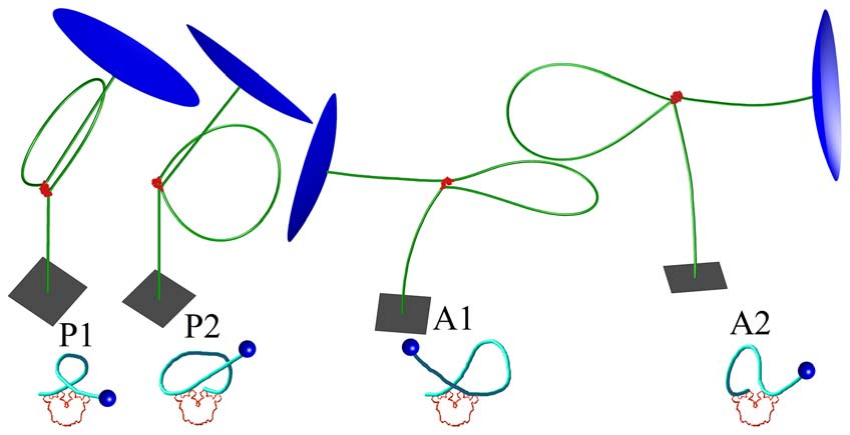
Minimum energy configurations of a 1632 bp segment in a TPM experiment. The 900 bp loop formed between binding sites is located at 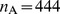, and 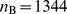. Lac repressor is shown in red, part of the attachment plate is shown as a gray square, and part of the 160 nm bead surface is shown in blue. The sketch of the binding topology for each loop type is depicted in a diagram below.

For the purpose of calculating looping probabilities associated with TPM experiments, we generate four different canonical ensembles: 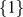 DNA configurations with no loops. 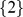 Configurations with “freely-jointed loops”, for which only the distance, 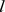, between the two loop termini (centers of binding sites) is held fixed. 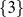 Configurations for which the barycenter of one loop terminus is fixed in position relative to the other terminus, but the terminus is free to have any orientation. 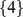 Configurations containing loops of prescribed topology, for which the two loop termini have fixed relative position and orientation and the loop has fixed linking number. (The linking number is a topological invariant equals to the number of times one of the strands of a circularized DNA molecule is linked with the other.) Since a loop is not a closed DNA segment, for the purpose of computing its topological and geometrical properties we close the looped segment by including the terminus-to-terminus step as an additional virtual base-pair step. The linking number associated with a loop is given by the relation
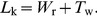
(22)(Precise definitions are given in [42] and [43].) We calculate the writhe, 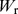, according to the scheme proposed in appendix B of [44] and the total twist (in turns), 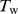, using the formula proposed recently by [45]. The loop with rigid V-shaped Lac repressor can be one of four types suggested in [12]: two parallel loop types P1 and P2, and the two antiparallel types A1 and A2. (See Figure 4). Although for each such type the relative orientation between the two base pairs associated with the loop termini is precisely defined, each group may contain multiple loops with topologies differing by the linking number.

When one has in hand 4 canonical ensembles of the conditions described above, one can use ensemble 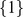 to calculate the probability 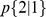 of having configurations obeying condition 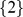 given that they obey condition 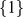. This is done by recording the number of configurations from ensemble 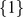 for which the distance 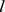 between the loop termini obeys 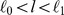. Similarly we denote 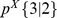 (or 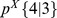) as the conditional probability of satisfying 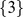 (or 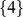) under 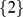 (or 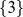) for loop type 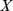. For the calculation of 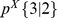 we used the ensemble 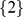 to record the number of configurations for which one loop terminus is within the volume of a cone that is fixed with respect to the other loop terminus and its vertex coincides with the center of that terminus. To estimate 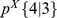 we use the axis-angle representation to calculate, for each configuration in ensemble 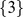, the angle of rotation, 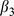, needed to bring one loop terminus to a relative orientation (with respect to the other terminus) that is equal to that of the specified loop type 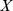. For this calculation one records the number of configurations in ensemble 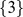 for which the angle of rotation is close enough to zero. Ensembles obeying condition 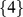 are used for calculations of the distribution of the measurable projected (onto the plate) end-to-end distance, 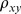, between the bead and the DNA end that is attached to the plate.

We here calculate the 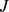-factor, 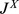, associated with a given loop type 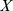 as follows:
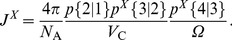
(23)Where 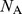 is Avogadro's number, and 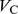 is the volume bounded by the surfaces of spheres of radii, 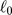 and 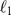, and a cone with vertex angle 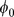 (with the centers of the spheres and the vertex of the cone in a coincidence.), i.e.,
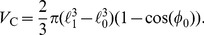
(24)The volume, 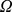, of the group of proper rotations is calculated using equation (21):
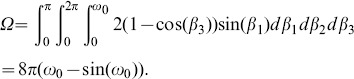
(25)For all the calculations reported here we used 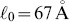, 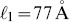, 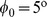, and 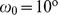. We found these values sufficiently small to be close enough to the limiting values in which 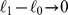, 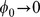, 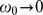, but in the other hand high enough so that the flexibility of the Lac repressor can be taken into account [27]. All ensembles consisted of 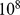 configurations.

**Figure 5:**
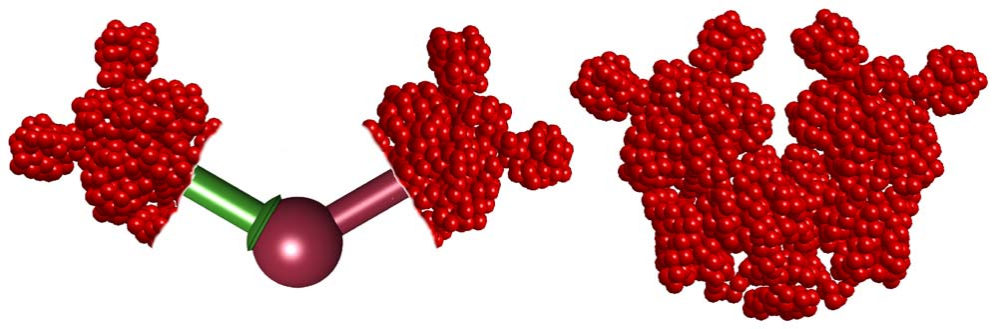
An illustrations of the Lac repressor in its possible conformations. A space filling model of the Lac repressor tetramer in its stiff V-shaped conformation (right) and a schematic representation of our assumed model of the open (extended) conformation of the Lac repressor (left). To simulate the open conformation, we assumed that the two dimeric arms of the Lac repressor are connected by a spherical joint that permits them to rotated freely about the joint. Thus, the three degrees of freedom characterizing the relative orientation between the two arms can attain any feasible value in the configurational space with no energetic cost for the conformational change of the Lac repressor.

We consider the possibility that when a Lac repressor is bound to both operators it may be either in its V-shape conformation or in an open conformation in which its two dimeric arms are open [46] [12]. To mimic the open conformation we regard the two dimeric arms of the Lac repressor as if they are connected through a spherical joint as shown in Figure 5. Accordingly, each arm is free to rotate (together with the confined lac-repressor head group) about the joint.

### Dependence of end-to-end distribution on Lac repressor concentration

For our calculations bearing on the dependence of the distribution of 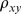 on the concentration, [LacI], of the tetrameric Lac repressor we follow the theory proposed by [19], [27]. In the TPM experiments discussed here, the DNA sequence includes two operator sequences (binding sites), O1 [47] and Oid. (Although *in vivo* looping occurs between operators O1 and O2 or O1 and O3, looping experiments generally utilize the ideal operator Oid, which binds much more tightly to the Lac repressor. The reason is that a loop with operator O2 or O3 has too brief a lifetime to be observed in a TPM experiment. The use of Oid alters the proportion of looped and unlooped configurations, and hence any interpretation to *in vivo* experiments must be done with care.)

The symmetric operator Oid [48], [49] was used to achieve higher binding affinity [50], resulting in increased durations of looping events. The consequent large difference between 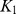 and 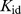 permits a simplified calculation of experimental loop probabilities. See e.g., equation 4 in [19]. This simplification has been used very recently in [51].

The binding affinities for these operators are characterized by the dissociation constants 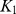 and 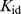. Since the binding affinity of the ideal operator Oid is significantly higher than that of the operator O1 we have 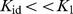. The interaction of such DNA with the Lac repressor can be classified into the following states:

1. Both of the operators are free.
2. One operator is bound:
  - O1 is bound while Oid is free.
  - Oid is bound while O1 is free.
3. Both operators are bound, each to a different Lac repressor.
4. Both operators are bound to the same Lac repressor and the resulting DNA loop is:
  - Type P1 with rigid V-shaped Lac repressor.
  - Type P2 with rigid V-shaped Lac repressor.
  - Type A1 with rigid V-shaped Lac repressor.
  - Type A2 with rigid V-shaped Lac repressor.
  - Type Open with extended and flexible Lac repressor.

The probabilities of all states, based on the analysis suggested in [19] are written here, with a modification accounting for the inclusion of the open loop:
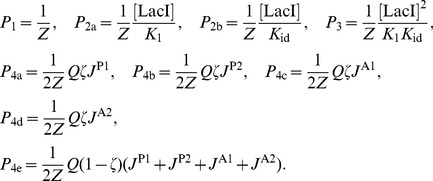
(26)Where the factor 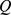 is given by
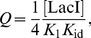
(27)and the number 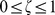 expresses the overall balance between the V-shape loops and the extended loops. In terms of the binding protein, 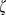 is the equilibrium ratio between its V-shaped conformation to its possible open conformation when bound to the two operators. This modification does not change the partition sum from the expression suggested in [19]. Therefore, 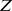 is given by:
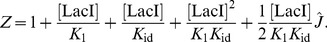
(28)Where 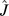 is the average 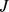-factor:
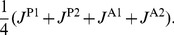
(29)


### Dependence of looping probability on phasing

**Figure 6:**
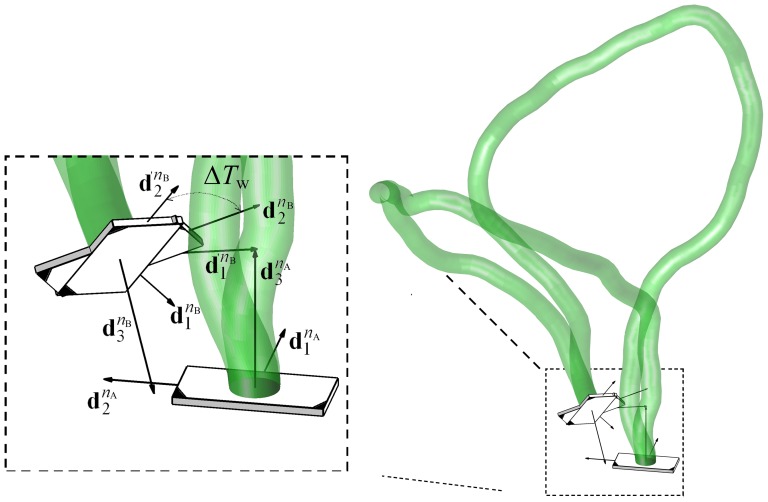
A schematic illustration showing two loops in an ensemble of type 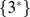. If one loop has its two termini in relative orientation and displacement that is in accord with one of the loop topological groups, and the second loop has its terminal base pair 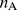 in coincidence with that of the first loop, the two termini of the loops at 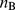 differ in their orientation by an angle 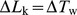 about 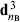.

To investigate the effect of changing the phasing between the loop termini, we calculate a canonical ensemble 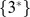 of configurations obeying three translational constraints but only two angular constraints. This means that the center of one loop terminus is fixed with respect to the other terminus, but that loop terminus is free to rotate about its unit vector 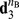, which is fixed in space in accord with the prescribed loop type. This ensemble permits us to analyze the dependence of 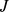-factor on the excess link, 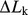, required to bring one loop terminus to a complete agreement with the orientation associated with the specified topology. An example of two configurations in this ensemble is schematically depicted in Figure 6.

Although a specified loop can be formed only with integral values of 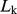, a canonical ensemble of the type 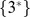 gives the probability distribution of a loop of a given type for any real value of 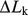.

For the calculation of the dependence of the 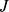-factor on 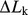, we modify the relation in (23) as follows:
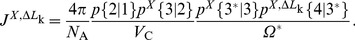
(30)The angular volume, 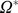, is given by
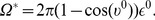
(31)The probability 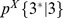 is calculated as the fraction of configurations in ensemble 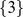 for which the angle, 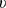, between 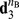 and 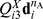 is such that 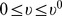. Note that equation (12) implies that the triads 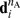 and 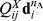 satisfy the loop end conditions associated with the topological group 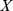. For the probability 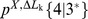 we count all configurations in ensemble 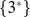 with excess link value within the interval 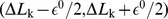. (See Figure 6).

### TPM Experiment

**Figure 7:**
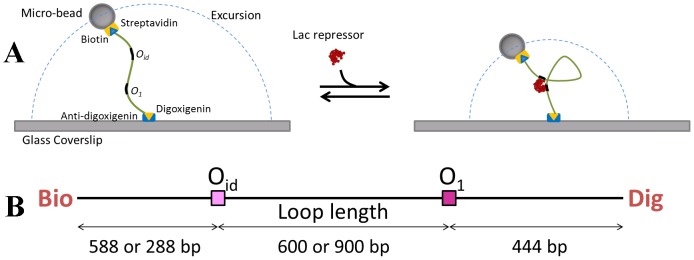
Illustrations of the TPM experiment and the 1632 bp DNA. **A**. DNA tether labeled with biotin and digoxigenin links a polystreptavidin-coated microsphere (bead) to an anti-digoxigenin coated coverslip. The motion of this tethered bead is characterized by its mean (or RMS) excursion, which exhibits a visible decrease when subject to the formation of Lac repressor mediated loop. **B**. Schematic linear representation of the 1632 bp DNA construct with Oid and O1 positioned 600 or 900 base pairs apart.

DNA fragments used in the TPM experiments, included the two Lac repressor operators, Oid (AATTGTGAGCGCTCACAATT) and O1 (AATTGTGAGCGGATAACAATT) sequences, spaced 600 or 900 bp apart (center-to-center distance). The protocol for the TPM experiments was similar to those published previously [52], [53]. Opposite ends of DNA tethers were labeled with biotin and digoxigenin to link a streptavidin-coated microsphere (bead) of radius 160 nm (Spherotech, Inc., Lake Forest, USA) to an anti-digoxigenin-coated coverslip (plate). The motion of beads in 10 mM Tris-HCl pH 7.4, 200 mM KCl, 5% DMSO, 0.1 mM EDTA, 0.2 mM DTT and 0.1 mg/ml 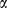-casein, with varying lac repressor concentrations (1 pM to 200 nM), was observed using differential interference contrast (DIC) microscopy. The experimental setup is shown schematically in Figure 7. The position of beads was tracked in real-time and recorded at 50 Hz with an exposure time of 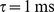. To remove instrumental drift affecting all beads in each field of view, positions were determined with respect to immobile beads in the same field of view. Asymmetric movement of the tethered bead is the simplest indicator of a bead attached to multiple tethers. To exclude these cases, any bead for which the scatter of observed positions displayed an ellipticity ratio greater than 1.07 was discarded from further analysis [54], [55].

**Figure 8:**
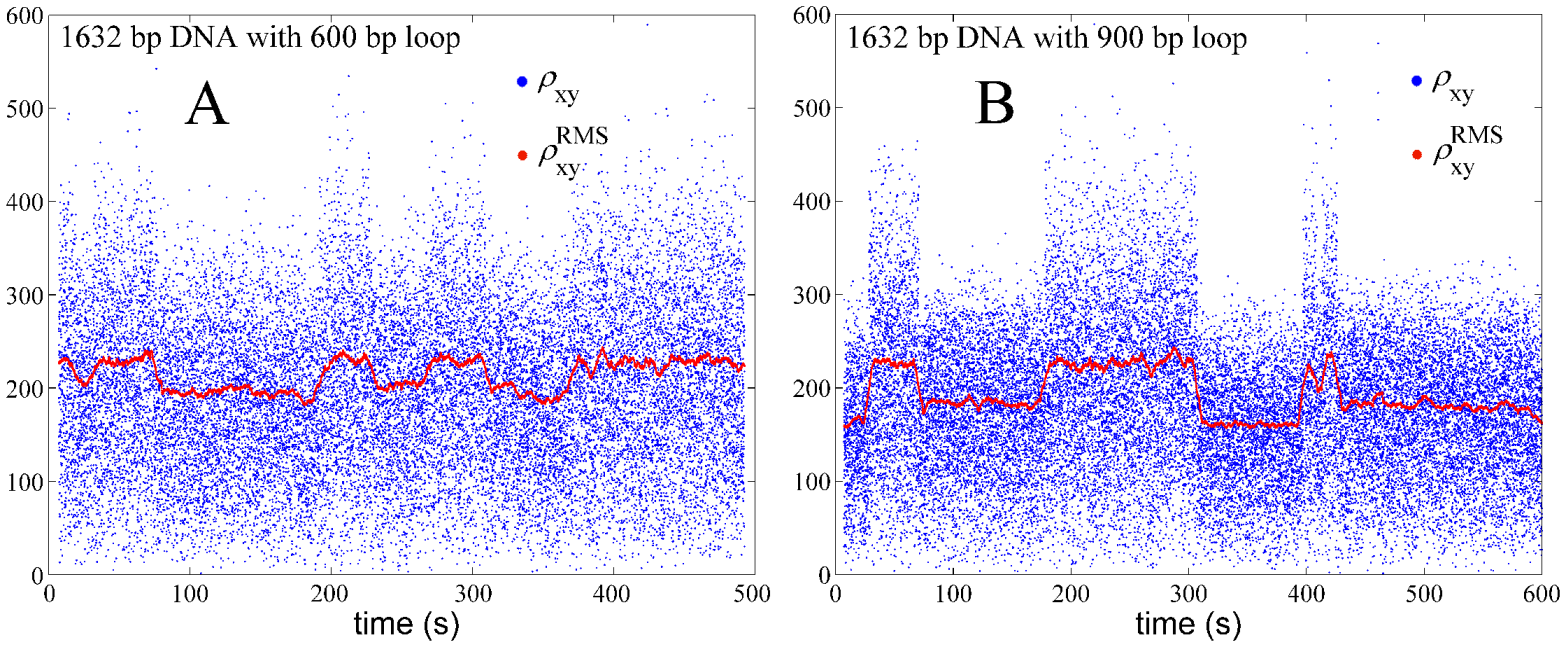
An example of our experimental results showing the projected end-to-end distance for experiments in which Lac repressor-induced loops were forming with length 600 (left) and 900 (right) bp. The blue dots show the projected end-to-end distance 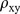 calculated using equation (32). The red dots, centered within each 8 s window, show the RMS values according to equation (33). Each pronounced drop in the red trace corresponds to a looping event.

The point of attachment was calculated for each time window as the barycenter of the 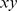 scatter that includes all the projected (onto the plate) positions of the bead center measured within the associated time window. For the results reported here, 8 second time windows were used. At each recorded time point 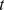, the excursion i.e., the 2D projected distance between the bead and the point of attachment, 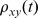 was determined using
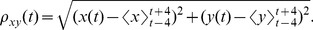
(32)The root mean square of the projected distance, 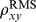, used as a measure of the excursion, was also averaged over 8 second window,
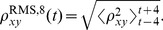
(33)Plots of the excursion with respect to time in Figures 8A and 8B reveal how the length of a single DNA tether changes during the course of the experiment due to the formation and breakdown of 600 bp and 900 bp loops, respectively. In each case the value of 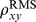 is close to one of two levels, the lower corresponding to looped configurations and the higher corresponding to unlooped tether configurations. Histograms of these values during observations of 20 to 50 beads for an aggregate time of 200–500 minutes were used to determine equilibria between looped and unlooped states for each loop size at a given Lac repressor concentration.

## Results

### Calibration curves

**Figure 9:**
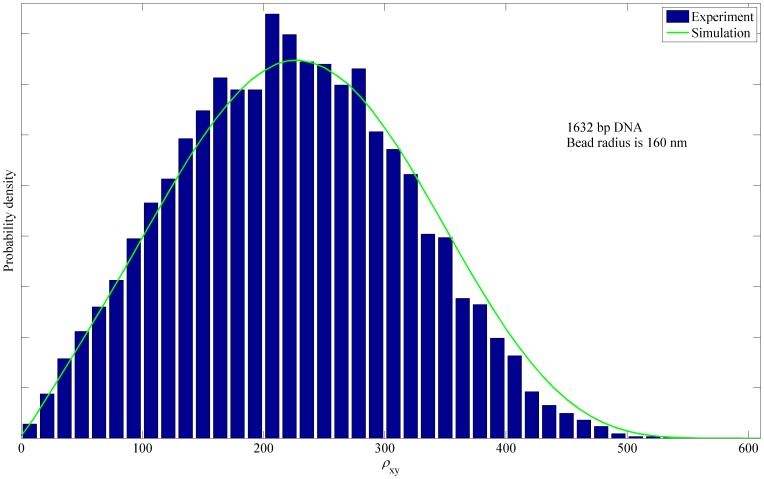
Experimental and simulated probability density of the projected distance for the 1632 bp DNA. The probability density of the projected distance 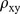 between attached DNA end and bead center, as measured in our TPM experiments (blue bars) and computed by numerical simulation (green curve). The 1632 bp tethered DNA molecule is unlooped for the duration of this experiment. The bead radius is 160 nm.

**Figure 10:**
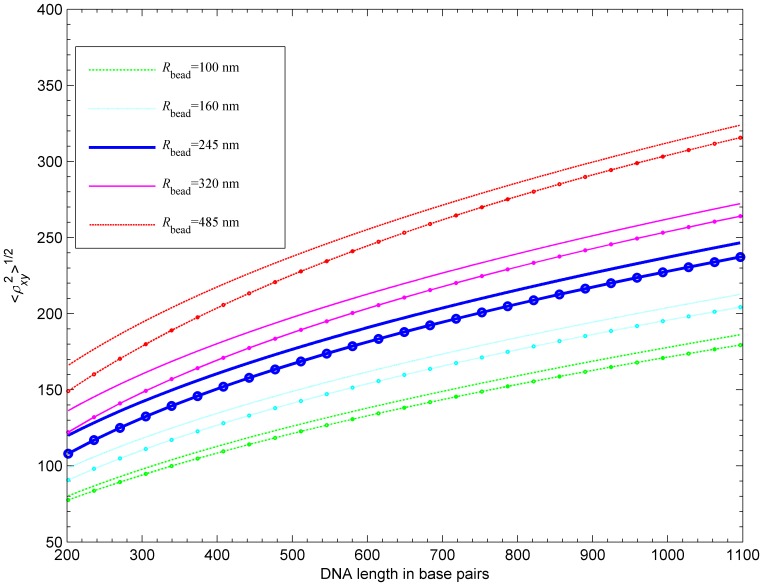
Calibration curves of the projected RMS distance (center of bead to attached end) for five bead radii. For each bead radius, a curve based on the numerical fit given in [26] is shown (marked with circles) together with our calculated curve. Our model is based on homogeneous DNA segments with persistence length of 476 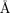.

A comparison between simulated and the experimentally determined probability distributions for projected bead center-to-tether end distance 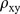 for the case of unlooped and protein-free 1632 bp DNA is shown in Figure 9. The largest differences between the theoretical and experimental distributions can be observed at the tail of the distribution (above 330 nm) where the theoretical prediction overestimates the measured distribution. The RMS value of the distribution (i.e., the quantity reported in Figure 10) is very sensitive to the tail of the distribution, which increases demands on the accuracy of simulation. Another potential issue is that the measured 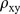 distribution utilizes the formula (32) which underestimates the true value of 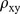 because it estimates the point of attachment by averaging over 8 s window.

Simulated calibration curves, i.e., curves showing the dependence of RMS value of the projected end-to-end distance between the center of the bead and the tethered end, 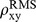 versus the tether length, are shown in Figure 10 together with experimental curves based on the numerical fit of collected data given in [26]. The results show that our predicted RMS value is greater by about 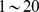 nm than the experimental results. Nelson et al. [26] found that lowering the persistence length to a smaller value (43 nm) leads to better agreement between the simulation and the data. However, the account of electrostatic repulsion and excluded volume in our simulations results in higher computed values of RMS than those reported in Nelson et al. [26] for the same persistence length (not shown). The discrepancy between our simulation data and the experiment is most likely due to discrepancy between the real experimental electrostatic screening and the value of ionic strength we assumed in the simulations. Other sources of discrepancy could be effects not accounted for in the simulation, such as the intrinsic curvature of the DNA tethers. The discrepancy decreases with the bead size and this decrease is more significant for short tethers. This suggests that the observed reduction of the in-plane motion of the bead could also be related to some form of attractive DNA-bead interaction, such as that caused by the hydrodynamic effects discussed in [56]. All of the discrepancy sources mentioned above are systemic and unlikely to affect relative positions of RMS value nor the ratios of 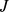-factors associated with different loop types studied in the next section.

### Dependence of radial distribution on Lac repressor concentration

Our simulations were focused on three cases for which data were available to us:

- 900 bp DNA molecule with the centers of the binding sites, O1 and Oid, located at 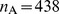, and 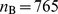 yielding 326 bp Lac repressor-induced loops,
- 1632 bp DNA molecule with the centers of the binding sites located at 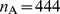, and 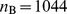 yielding 600 bp Lac repressor-induced loops, and
- 1632 bp DNA molecule with the centers of the binding sites located at 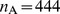, and 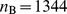 yielding 900 bp Lac repressor-induced loops.

The case **A** mimics the molecule investigated experimentally in [19], while the cases **B** and **C** correspond to molecules studied in our lab. To match the experimental setup, for the simulation of the 900 bp molecule we assumed a bead radius of 245 nm, while for the 1632 bp molecules the bead radius was taken to be 160 nm.

**Figure 11:**
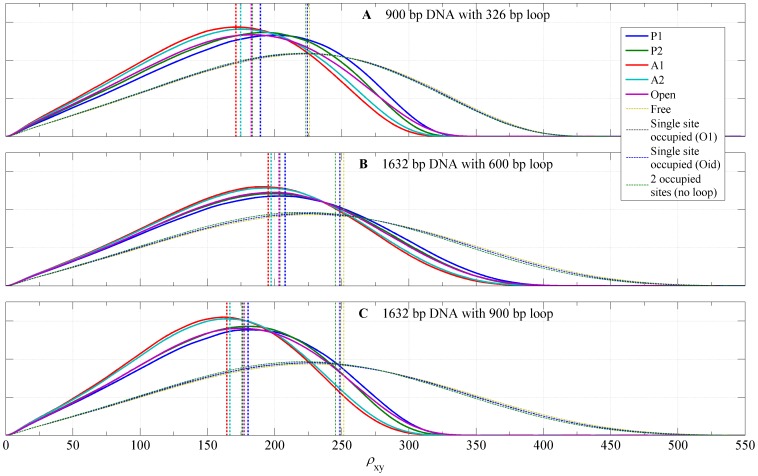
Probability density functions for the projected distances. The calculated probability density functions for 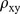, the projected distance between the DNA attachment point and the center of bead, for the loop topologies, P1, P2, A1, A2, the loop associated with the open conformation of the Lac repressor, an unlooped DNA with one or both sites occupied, and the free DNA. (**A**) 900 bp DNA attached to a bead with radius = 245 nm; loop length is 326 bp. (**B**) 1632 bp DNA attached to a bead with radius = 160 nm; loop length is 600 bp. (**C**) 1632 bp DNA attached to a bead with radius = 160 nm; loop length is 900 bp. The RMS value for each probability density is plotted as a vertical line.

Figure 11 shows computed distributions of the projected end-to-end distance, 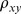, for DNA tethers with V-shaped loops of type A1, A2, P1, or P2 and with extended Lac repressor loops. In addition, the figure shows calculated distributions of 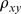 for tethers in which loop is not formed but one or both of the two binding sites are occupied. We note that distributions for these unlooped bound states of the DNA are different from the distribution for unlooped unbound DNA as the result of a bending of a single DNA operator when bound to a dimeric arm of the Lac repressor (about 60 degrees). The RMS values for each loop type are reported in Table 1. We found that, in each case, antiparallel loop type distributions have the smallest RMS values, followed by the parallel loops and the open loop. Not surprisingly, the largest RMS values were found for unlooped configurations, with RMS value increasing with the decreasing number of bound Lac repressor molecules in all three cases. The antiparallel loops have the smallest RMS distance because the angle between DNA exiting and entering the loop is about 120 degrees, while in parallel loops this angle is about 30 degress (see Figure 4). The variability of RMS distances for looped configurations is about 20 nm for case 900 bp DNA with 326 bp loop, less in the other cases, which is roughly 12% of the RMS distance of free DNA, large enough to be observable by TPM experiment.

**Table 1:** Calculated RMS values.

		A	B	C
Looped	A1	171.2	195.3	164.5
	A2	174.8	197.4	166.7
	P1	189.5	207.9	180.3
	P2	183.3	203.9	175.9
	Open	182.7	203.4	177.4
Unlooped	Oid occupied	223.3	245.2	245.3
	O1 occupied	224.4	249.0	249.0
	O1 & Oid occupied	224.5	248.5	248.1
	Free DNA	226.0	251.5	251.5

Calculated values of RMS (in nm) for individual state distributions shown in Figure 11. Cases: **A** (900 bp DNA with 326 bp loop and 245 nm bead), **B** (1632 bp DNA with 600 bp loop and 160 nm bead), and **C** (1632 bp DNA with 900 bp loop and 160 nm bead).

**Figure 12:**
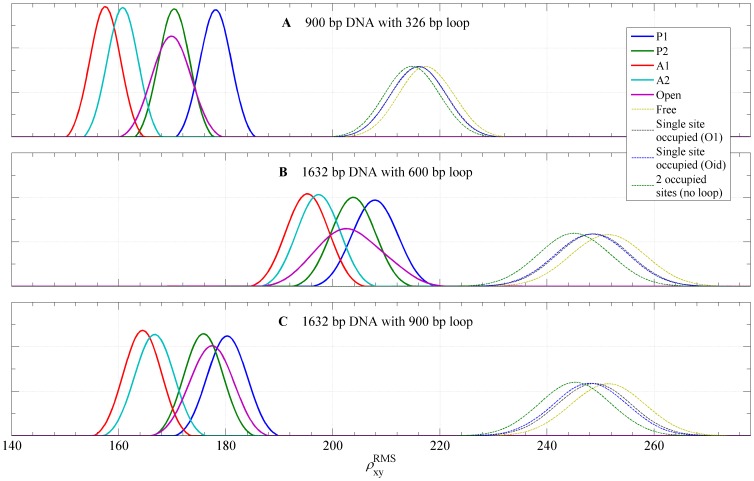
Probability density functions for RMS values of the projected distances. The probability density functions for RMS values of the projected distance calculated for the loop topologies, P1, P2, A1, A2, the loop associated with the open conformation of the Lac repressor, an unlooped DNA with one or both sites occupied, and the free DNA. (**A**) 900 bp DNA attached to a bead with radius = 245 nm; loop length is 326 bp. (**B**) 1632 bp DNA attached to a bead with radius = 160 nm; loop length is 600 bp. (**C**) 1632 bp DNA attached to a bead with radius = 160 nm; loop length is 900 bp. Each graph shows the distribution of 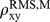 values (in nm), computed from 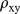 distributions in order to mimick the window averaging of 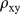 traces during the processing of TPM traces (see text).

Although the results in Figure 11 contain the complete information about the distribution 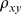 for looped DNA, these probability distributions cannot be compared directly to experimental TPM results as they are usually reported. In TPM experiments, the projected position of the bead is recorded in a rate of about 30–50 frames per second and reported as the distribution of the root mean square value 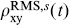 averaged over frames taken within a time window of 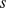 seconds, as in equation (33). In the TPM experiments reported in [19], 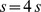 and hence, each RMS value is calculated from about 120 consecutive values of 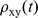. (In our experimental results for the 1632 bp molecules, we found it more instructive to double the size of the time window to 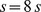.) In order to obtain a distribution to compare with the experimental windowed distribution we compute the distribution of 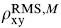, which is the RMS of 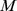 randomly chosen 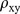 values from the original ensemble. The number 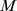 (here chosen to be 120 for the case **A** and 200 for **B** and **C**) is the simulation equivalent to the length of the time window. Computed distributions of 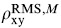 for each looped and unlooped DNA are shown in Figure 12. In addition to the windowing, it was reported in [19], [27] that their TPM experiments were underestimating the true RMS value due to the blurring of the image of the bead caused by a long exposure time 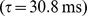 in a single frame taken in the TPM experiments. For case **A**, which is to be compared with experimental results of [19] we took the blurring into account in computing the distribution 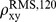. For our experiments the exposure time is 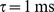, which eliminates blurring effect. (See the discussion in the supplementary information provided in [27]). With this transformation the separation of different loop types is much more apparent. Note that in each case the distribution corresponding to open Lac repressor conformation is wider than distributions corresponding to V-shaped loop types, due to the less stringent constraints on the bound loop resulting from the flexibility of the protein.

Simultaneously with calculation of the distributions we computed the 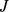-factors for each loop type using the scheme discussed in the subsection on the statistical mechanics. The values of 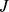-factors for different loop types are reported in Table 2.

**Table 2:** Calculated values characterizing looping probabilities.

	A	B	C
*J* ^P1^	4.0 nM	5.13 nM	1.05 nM
*J* ^P2^	3.6 nM	6.08 nM	1.56 nM
*J* ^A1^	11.5 nM	6.75 nM	3.59 nM
*J* ^A2^	16.8 nM	20.51 nM	10.28 nM
*K*_1_	1.00 nM	0.86 nM	0.86 nM
*K*_id_	1.47 pM	5.13 pM	5.13 pM
*ζ*	0.70	0.31	0.57

Calculated values of 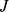-factors, and optimized values of binding constants 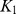, 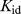, and the open loop ratio 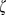 for the three cases described in the text: **A** (900 bp DNA with 326 bp loop and 245 nm bead), **B** (1632 bp DNA with 600 bp loop and 160 nm bead), and **C** (1632 bp DNA with 900 bp loop and 160 nm bead). The dissociation constants 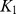, 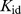, and the ratio 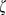 were obtained by performing an optimized nonlinear fit between the experimental results and theoretical joint distribution as function of [LacI] (see equation (26)) as described in the text. For the case **A** we used experimental results shown in Figure 3 of [19].

Once all simulations were concluded, we used the calculated values for the 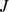-factors, Equations 26, and assumed values of the binding constants 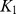, 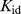, and the Lac repressor opening ratio 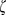 to predict the joint distribution of 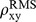 as a function of the concentration [LacI] of the Lac repressor. We then optimized the 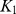, 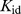, and 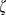 to obtain the closest fit between the relative occupancies of various looped states in our theoretical prediction and the corresponding values obtained experimentally. The optimal values of the parameters 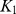, 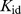, and 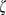, for all three cases are given in Table 2.

**Figure 13:**
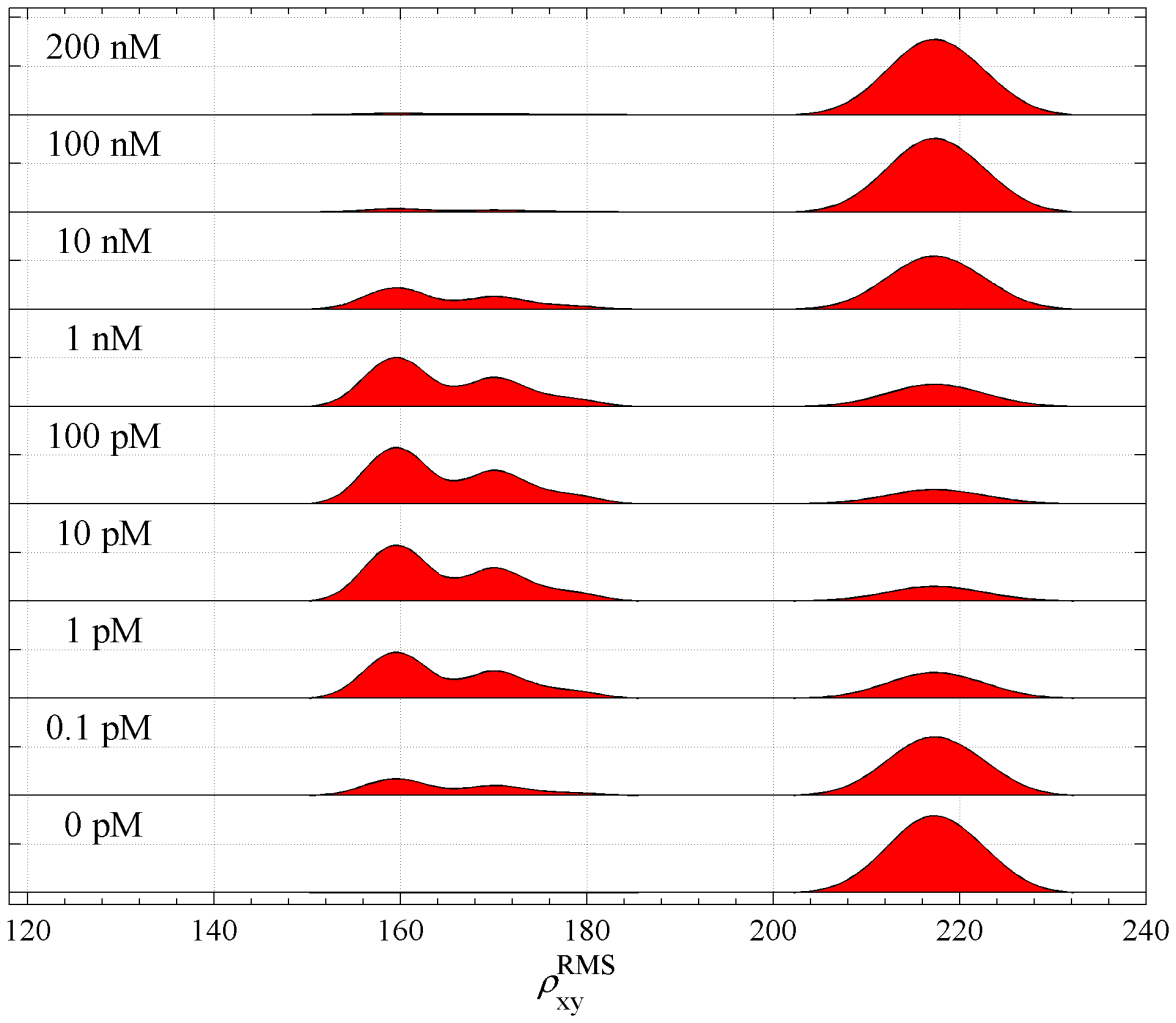
The predicted joint distribution of RMS projected distances as a function of the concentration of Lac repressor for the 900 bp DNA. The predicted joint distribution of 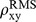 was calculated from the probability density functions given in Figure 12A, and our calculated looping 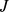-factors. Here the bead radius is 245 nm, the DNA length is 900 base pairs, the centers of the binding sites are located at 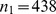, and 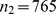 yielding 326 bp Lac repressor-induced loops.

Resulting optimized distribution for case **A** is shown in Figure 13, which is to be compared with Figure 3 of [19]. The fitted distribution was able to recover qualitative and quantitative features of the experimental distribution, namely, the decomposition of the distribution into one double-peaked component corresponding to a mixture of looped molecules and one single-peaked component corresponding to a mixture of unlooped states. In the double-peaked component, the peak with smaller RMS peak value corresponds to a mixture of loops of types A1 and A2, while the peak with larger RMS corresponds to a mixture of loops of types P1, P2 and the open Lac repressor loop. With increasing Lac repressor concentration the percentage of looped molecules increases until about 10 pM of Lac repressor and then decreases until it vanishes above 100 nM of Lac repressor. This same behavior is seen in the experiments. The distance of the peaks in the double-peaked component is about 10 nm, which is smaller than 25 nm seen in the experimental data. The distance between the outermost peaks in the theoretical distribution is about 58 nm, which is identical to the experimental distribution. The RMS values of peaks in the theoretical distribution are about 20 nm higher than in the experimental, in accord with the discrepancy observed for the calibration curve. In summary, our theoretical approach correctly predicts the number and relative heights of peaks in the RMS distribution, but not their absolute positions.

**Figure 14:**
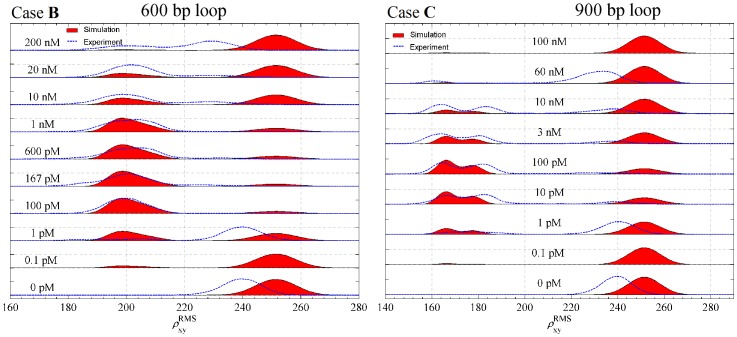
The predicted joint distribution of RMS projected distances as a function of the concentration of Lac repressor for the two cases of the 1632 bp DNA. Computer simulation and Experimental TPM results showing a DNA molecule of 1632(Case **B**) 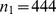, and 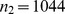 yielding 600 bp Lac repressor-induced loops; and (Case **C**) 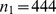, and 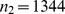 yielding 900 bp Lac repressor-induced loops. The bead radius for both cases is 160 nm. The figure shows the joint distribution of RMS projected distance between the center of the bead attached to the end for which 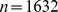 to the DNA end that is attached to the plate. Each single RMS value in the experimental results (blue) was calculated using a time window of 8 s. The simulation results (red) were calculated from the probability density functions given in Figures 12B, and 12C and our calculated looping 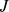-factors (See Table 2).

In a similar fashion we fitted the theoretical predictions for cases **B** and **C** to our TPM results for the two 1632 bp molecules. We have estimated the values of parameters 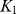, 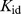 (assuming they are identical for the two cases since the experiments were performed in identical conditions), and the parameter 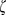 for each case. The resulting computed joint distributions for various concentrations of Lac repressor are shown in Figure 14 plotted next to the experimental results. The computed values of 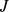-factors and optimized values of the parameters 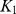, 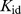, and 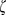, are again reported in Table 2. The qualitative features of the distributions are the same as in the case **A** with minor differences. The double-peaked component of the distribution in the case **B** is not as pronounced as in the case **A**, because the RMS values for individual loop types are closer together and their distributions of 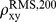 are wider than those in case **A**, as a result of a longer tether (1032 bp in the case **B** compared to 574 bp in the case **A**). The case **C**, for which the looped tether length is 732 bp, is intermediate between **A** and **B**. As shown in Figure 14, for this case, the double peak is visible but not as pronounced as in the case **A**. As in the case **A**, our simulations recover the relative heights of the experimentally determined looped and unlooped distributions as function of Lac repressor concentration for almost all concentrations. In the case **B** our simulations are overestimating (compared to the experimental results) the percentage of unlooped configurations at Lac repressor concentrations of 10 nM and 20 nM, while in the case **C** the simulations are overestimating the percentage of unlooped configurations at Lac repressor concentrations of 3 nM and 10 nM.

The key component contributing to the occupancy of the middle peak of the distribution in the cases **A** and **C** is the loop with extended Lac repressor. Equation (26) implies that the ratio 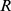 of occupancies of the middle peak (consisting of loop types P1, P2, and Open) and lower peak (consisting of loop types A1 and A2) is independent of the binding constants 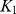, 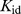 and the concentration [LacI] of the Lac repressor, and is given by
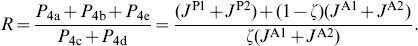
(34)Note that if the Open loops (with extended Lac repressor) were absent from the ensemble (i.e., if 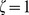 in the above equation) then, in view of the 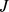-factors reported in Table 2, in the case **A**, 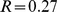, which is smaller than the average value 0.46 observed in Han et al. [19]. In the case **C**, the computed value 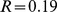 for 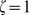 is also significantly smaller than the value 1.09 estimated from experimental distribution in Figure 14, case **C**. Therefore, in the absence of extended Lac repressor loops the occupancy of the middle peak in the distributions would have to be substantially lower than what the data show.

### Dependence of radial distribution on phasing

The 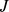-factor of a DNA loop is very sensitive to phasing, i.e., the amount of excess link trapped in the loop upon closure. This sensitivity can be explored by changing the loop length by 1 bp at a time - such a change has little effect on the loop length but a large effect on excess link due to the intrinsic helicity of DNA which has a period of about 10.5 bp [57], [58], [59]. TPM experiments of this type were performed by Han et al. [19] with DNA constructs of length 900–903 bp containing O1 and Oid sites separated by any chosen distance in the range 300–310 bp. Each change in the loop length by 1 bp results in a change in the excess link of the loop of about 1/10 of a full turn. The change in the looping probabilities is clearly visible in the experimental joint distribution (Figure 7 of [19].)

**Figure 15:**
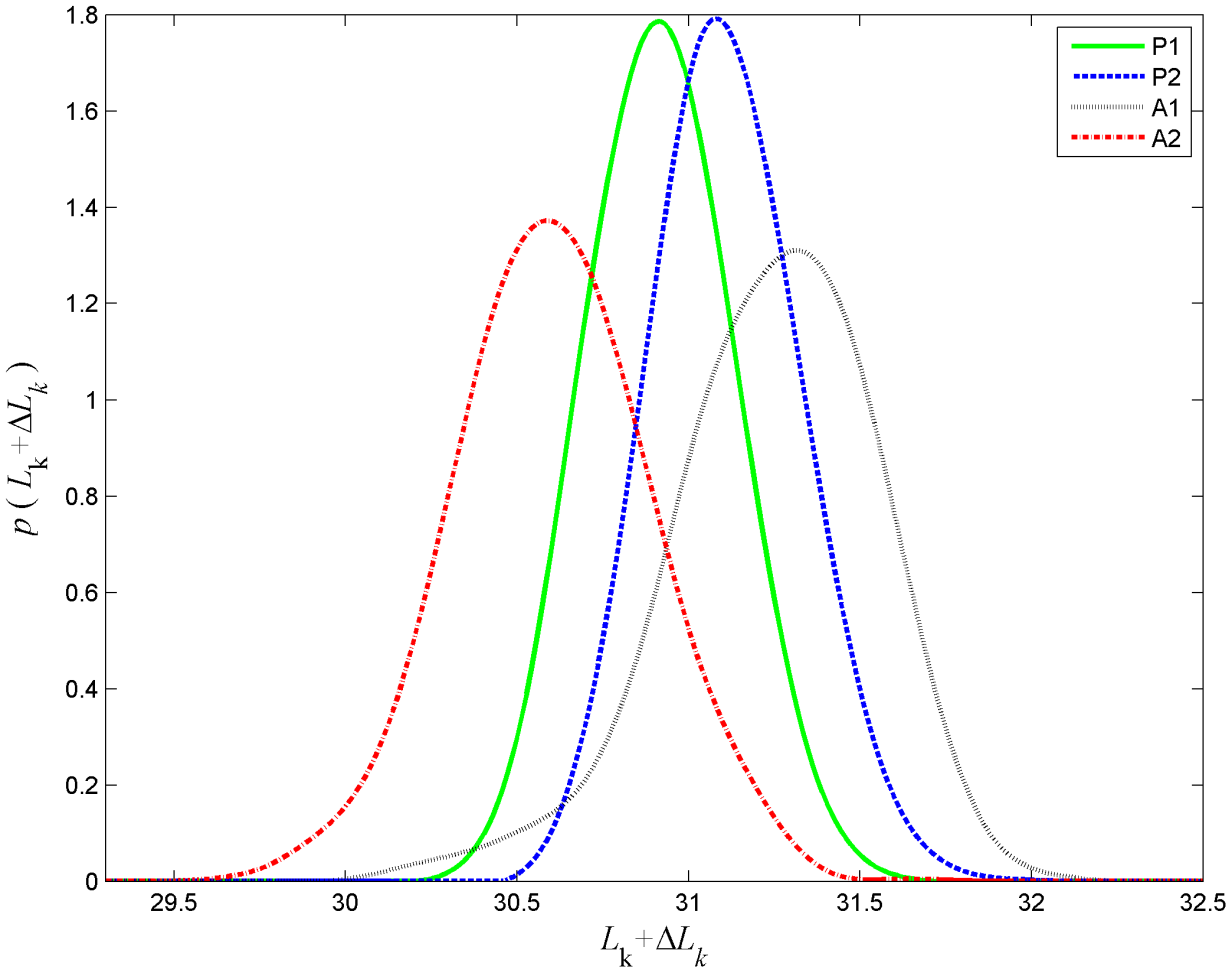
Probability distribution function for the excess link values associated with each of the loop types. The probability density function of various topoisomers for the loop topologies, P1, P2, A1, and A2 for simulated 900 bp DNA with 326 bp loop length and bead radius of 245 nm.

We here model the effect of changing the excess link of a loop by using the approach described in Methods section. In particular, we constrain the position and tangent orientation of one of the bound DNA operators but allow it to rotate around the tangent. The angle of rotation divided by 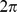 describes the change of the linking number (or, correspondingly, the excess link) of the loop. Although a specified loop can form only with integral values of 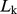 a canonical ensemble of the type 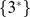 gives, for a given loop type, the probability distribution of 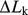 from which the most likely linking number for that loop type can be deduced. Our simulation results of the distributions for each loop type of the 900 bp molecule are shown in Figure 15. The interpretation of results depends on the loop length. For loop of length 326 bp (original loop length in the simulation) only topoisomers with integer values of 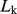 are possible and our results indicate that the loops of type P1 and P2 with linking number 31 are the most likely to be formed. For the loop type A1 the vast majority of loops has linking number 31, while a small fraction of loops of this type form with linking number 32. Our results show that the loops of type A2, which has the highest probability to form overall, i.e., for which the 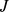-factor is the largest, may appear with two competing linking numbers 30 or 31. When the results are to be interpreted for a different loop length, say 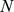 bp, then the resulting topoisomers have linking numbers shifted by 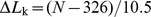. For example, for 321 bp loop 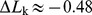 and hence loops with linking numbers 30.52 and 31.52 would appear in the mixture. In this case for loops of type A1 vast majority would have linking number 30.52 while loops of type A2 would all have linking number 31.52. Loops of types P1 and P2 would split between the two alternatives.

**Figure 16:**
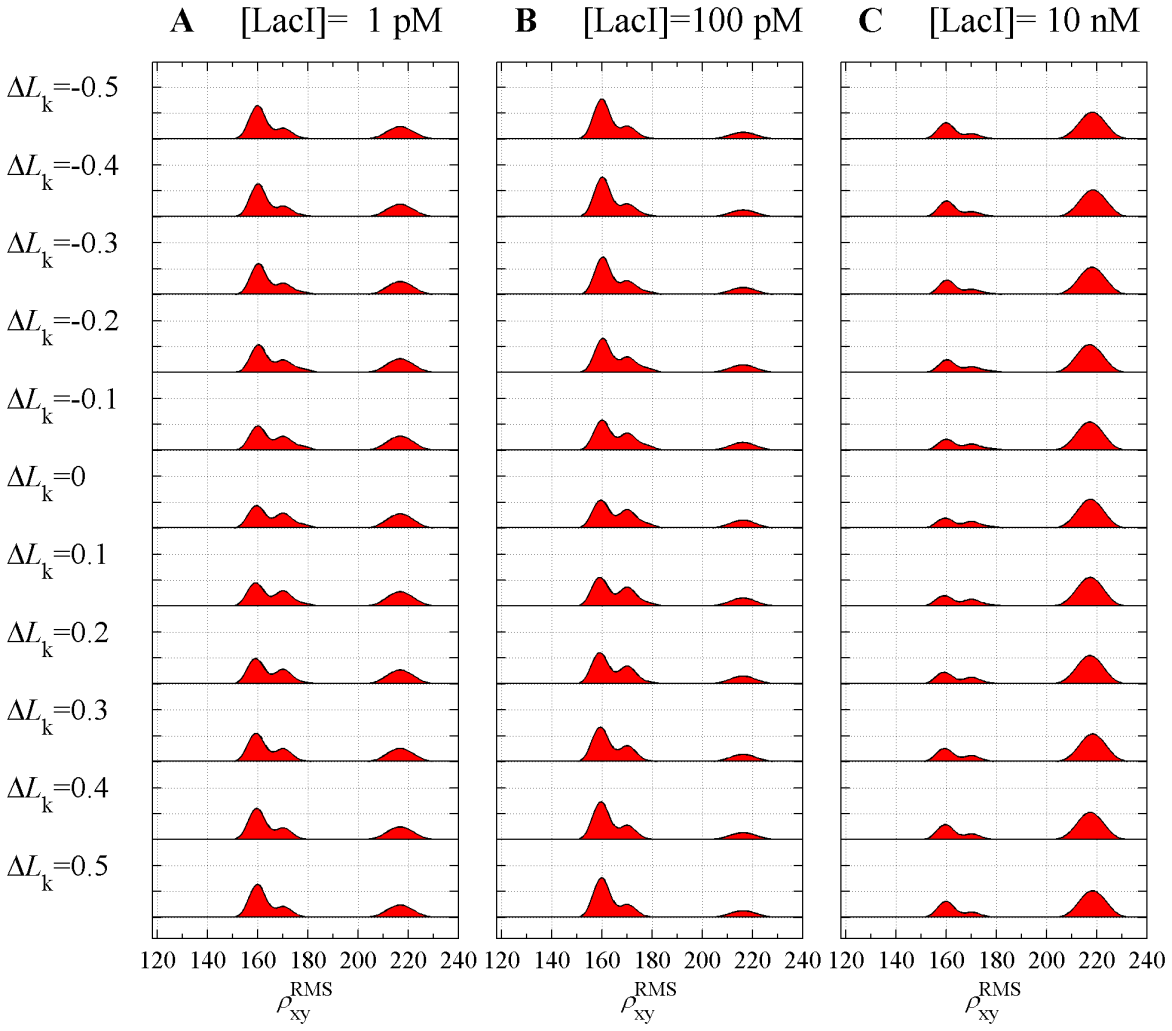
Joint distribution of RMS as a function of the excess link for three concentrations. The predicted joint distribution of RMS projected distances, 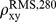, is shown as a function of the excess link, 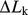 for the 900 bp DNA. The distribution was calculated from the probability density functions given in Figures 12A and 15, and the looping 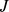-factors. For each value of 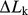 all configurations with values of 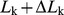 differing by an integral number were taken into account.

Using equation (30), the probability density function of 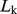 (Figure 15), and the probability density of the projected end-to-end distance (Figure 12A) we computed the RMS distributions corresponding to excess link values shown in Figure 16. In this figure one can see the variability in the proportion of peak heights in the portion of the distribution corresponding to looped configurations. The antiparallel loops A1 and A2, comprising the left-most peak, are of the highest percentage when the linking number differs by about half a turn from an integer value, which is in agreement with the experimental results reported in [19].

## Conclusions

We have introduced a novel numerical scheme for accurate statistical mechanical simulation of constrained DNA molecules, and used that scheme to compute the RMS distributions reported in TPM studies of DNA looping induced by the Lac repressor. Our modeling scheme enhances the interpretation of TPM experiments by associating geometrical and topological information to the peaks observed in the experimental TPM distribution of RMS bead-to-attachment site distance. Our results strongly suggest that the looped states observed in the experimental TPM distribution are, in fact, composed of five distinct looping states. The peak with lower RMS value corresponds to the antiparallel types A1 and A2 while the peak with higher RMS value corresponds to the parallel types P1 and P2 and a looped state in which Lac repressor attains an open, flexible conformation. The main reason that necessitates the inclusion of the open state is that without it, the parallel types P1 and P2 do not occur with high enough probability to explain the observed area of the peak.

Besides the structure of the looped states, we have used the model to estimate the Lac repressor-DNA operator dissociation constants. The ranges of our estimates, 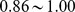 nM for 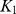 and 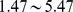 pM for 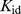 compare well with the ranges obtained by Han et al [19] by a similar technique (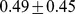 nM for 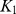 and 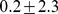 pM for 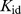). Our estimated range for 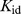 is within the range of values measured using binding assays (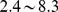 pM [60]) while our estimates for 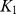 are about 50 times higher than bulk binding assay results (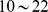 pM [60]) which would correspond to 50-fold weaker binding. It is important to note that no DNA looping occurs in such binding assay experiments and therefore the conditions for DNA-protein binding are close to ideal. In the TPM experiments, on the other hand, the DNA deformation that leads to the formation of loops may also result in local deformation of the DNA binding site for Lac repressor which would lower the DNA-protein binding affinity (increase the dissociation constant). Such an effect is difficult to quantify as we do not possess at the moment an accurate theory of the dependence of DNA-protein binding affinity on local DNA deformation.

We have generated an arsenal of large canonical ensembles of configurations confined to an appropriate set of geometric constraints, and use this arsenal to calculate the looping 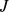-factors characterizing looping probabilities as the product of conditional probabilities in a manner similar to that utilized in [24]. In contrast to the approach in which all the conditional probabilities are taken from a small fraction of a single canonical ensemble calculated using Gaussian sampling method [24], [27], [19], in our present scheme each of the conditional probabilities is deduced from an adequate ensemble that is generated separately.

In our numerical studies we calculate canonical distributions of constrained DNA molecules. In contrast to other simulations of looped DNA such as those reported in [19], [27] in which the fluctuations of the looped subsegment are neglected by either treating it as rigid or as a single extended step, in our approach fluctuations of the loop itself are taken into account. We believe that because of the loop-DNA steric effects, and the fact that all other intramolecular excluded volume effects and electrostatic interactions are taken into account, our results overestimate the mean (or RMS) end-to-end distances when compared to either experimental results, or other simulations in which some or all intramolecular interactions are not taken into account. This may suggest, that not only the bead volume exclusion effects that were analyzed in [61] and were taken into account in this work, are important, but also hydrodynamic effects [56] and possible steric attraction effects between the large bead and the DNA molecule may play a significant role and should be treated carefully in future models. Nonetheless, our results show qualitative agreement with both our experimental data and the results reported in [19] and support the existence of extended conformation of the Lac repressor.

Our simulations suggests that the looping probabilities for the anti-parallel loop types are 4- and 6-fold higher than those of the parallel loop types, even for loops of length 600 and 900 base pairs. This is somewhat surprising, as one expects the orientations of the two loop termini to be uncorrelated for loops significantly longer than the persistence length of the DNA. However, we believe, this is a direct result of the TPM experimental design. Because the total length of the DNA is kept unchanged in our experiments, a longer loop makes the upstream operator closer to the comparatively large bead. Consequently, the orientation of that operator is more correlated with the, very restricted, position of the bead. This makes the anti-parallel loops entropically favored.

## References

[1] Matthews KS (1992) DNA looping. Microbiological Reviews 56: 123–136. 1579106 10.1128/mr.56.1.123-136.1992 PMC372857

[2] Schleif R (1992) DNA looping. Annual Review of Biochemistry 61: 199–223. 10.1146/annurev.bi.61.070192.001215 1497310

[3] Müller-Hill (1996) The Lac Operon. Berlin

[4] Oehler S, Eismann ER, Krämer H, Müller-Hill B (1990) The three operators of the Lac operon cooperate in repression. The EMBO Journal 9: 973–979. 2182324 10.1002/j.1460-2075.1990.tb08199.x PMC551766

[5] Becker NA, Peters JP, Lionberger TA, Maher LJ (2013) Mechanism of promoter repression by Lac repressor DNA loops. Nucleic Acids Research 41: 156–166. 23143103 10.1093/nar/gks1011 PMC3592455

[6] Finzi L, Gelles J (1995) Measurement of lactose repressor-mediated loop formation and breakdown in single DNA molecules. Science 267: 378–380. 7824935 10.1126/science.7824935

[7] Schafer DA, Gelles J, Sheetz MP, Landick R (1991) Transcription by single molecules of RNA polymerase observed by light microscopy. Nature 352: 444–448. 1861724 10.1038/352444a0

[8] Yin H, Landick R, Gelles J (1994) Tethered particle motion method for studying transcript elongation by a single RNA polymerase molecule. Biophysical Journal 67: 2468–2478. 7696485 10.1016/S0006-3495(94)80735-0 PMC1225632

[9] Geanacopoulos M, Vasmatzis G, Zhurkin V, Adhya S (2001) Gal repressosome contains an antiparallel DNA loop. Nature Structural Biology 8: 432–436. 11323719 10.1038/87595

[10] Friedman A, Fischmann T, Steitz T (1995) Crystal structure of Lac repressor core tetramer and its implications for DNA looping. Science 268: 1721–1727. 7792597 10.1126/science.7792597

[11] Ruben GC, Roos TB (1997) Conformation of Lac repressor tetramer in solution, bound and unbound to operator dna. Microscopy research and technique 36: 400–416. 9140942 10.1002/(SICI)1097-0029(19970301)36:5<400::AID-JEMT10>3.0.CO;2-W

[12] Swigon D, Coleman BD, Olson WK (2006) Modeling the Lac repressor-operator assembly: The inuence of DNA looping on Lac repressor conformation. Proceedings of the National Academy of Sciences 103: 9879–9884. 10.1073/pnas.0603557103 PMC1502547 16785444

[13] Bond LM, Peters JP, Becker NA, Kahn JD, Maher LJ (2010) Gene repression by minimal Lac loops in vivo. Nucleic acids research 38: 8072–8082. 21149272 10.1093/nar/gkq755 PMC3001091

[14] Hirsh AD, Lillian TD, Lionberger TA, Perkins N (2011) DNA modeling reveals an extended Lac repressor conformation in classic in vitro binding assays. Biophysical journal 101: 718–726. 21806940 10.1016/j.bpj.2011.06.040 PMC3145271

[15] Mehta RA, Kahn JD (1999) Designed hyperstable Lac repressor: Dna loop topologies suggest alternative loop geometries. Journal of molecular biology 294: 67–77. 10556029 10.1006/jmbi.1999.3244

[16] Edelman LM, Cheong R, Kahn JD (2003) Fluorescence resonance energy transfer over 130 basepairs in hyperstable Lac repressor-dna loops. Biophysical journal 84: 1131–1145. 12547794 10.1016/S0006-3495(03)74929-7 PMC1302690

[17] Haeusler AR, Goodson KA, Lillian TD, Wang X, Goyal S, et al (2012) FRET studies of a landscape of Lac repressor-mediated DNA loops. Nucleic acids research 40: 4432–4445. 22307389 10.1093/nar/gks019 PMC3378866

[18] Vanzi F, Broggio C, Sacconi L, Pavone FS (2006) Lac repressor hinge exibility and DNA looping: single molecule kinetics by tethered particle motion. Nucleic Acids Research 34: 3409–3420. 16835309 10.1093/nar/gkl393 PMC1524907

[19] Han L, Garcia HG, Blumberg S, Towles KB, Beausang JF, et al (2009) Concentration and length dependence of DNA looping in transcriptional regulation. PLoS ONE 4: e5621. 19479049 10.1371/journal.pone.0005621 PMC2682762

[20] Swigon D (2009) The mathematics of DNA structure, mechanics, and dynamics. In: Benham C, editor, Mathematics of DNA Structure, Function and Interactions, Springer, Berlin. p. 293320.

[21] Benham CJ (1977) Elastic model of supercoiling. Proceedings of the National Academy of Sciences 74: 2397–2401. 10.1073/pnas.74.6.2397 PMC432179 267934

[22] Shimada J, Yamakawa H (1984) Ring-closure probabilities for twisted wormlike chains. application to DNA. Macromolecules 17: 689–698.

[23] Coleman BD, Olson WK, Swigon D (2003) Theory of sequence-dependent DNA elasticity. J Chem Phys 118: 7127–7140.

[24] Czapla L, Swigon D, Olson WK (2006) Sequence-dependent effects in the cyclization of short DNA. Journal of Chemical Theory and Computation 2: 685–695. 26626674 10.1021/ct060025+

[25] Swigon D, Olson KW (2008) Mesoscale modeling of multi-proteinDNA assemblies: The role of the catabolic activator protein in Lac-repressor-mediated looping. International Journal of Non-Linear Mechanics 43: 1082–1093. 23874000 10.1016/j.ijnonlinmec.2008.07.003 PMC3715064

[26] Nelson PC, Zurla C, Brogioli D, Beausang JF, Finzi L, et al (2006) Tethered particle motion as a diagnostic of DNA tether length. The Journal of Physical Chemistry B 110: 17260–17267. 16928025 10.1021/jp0630673

[27] Towles KB, Beausang JF, Garcia HG, Phillips R, Nelson PC (2009) First-principles calculation of DNA looping in tethered particle experiments. Physical Biology 6: 025001. 19571369 10.1088/1478-3975/6/2/025001 PMC3298194

[28] Biton YY, Coleman BD, Swigon D (2007) On bifurcation of equilibria of intrinsically curved, electrically charged, rod-like structures that model DNA molecule in a solution. J Elasticity 87: 187–210.

[29] Vologodskii AV, Levene SD, Klenin KV, Frank-Kamenetskii M, Cozzarelli NR (1992) Conformational and thermodynamic properties of supercoiled DNA. Journal of Molecular Biology 227: 1224–1243. 1433295 10.1016/0022-2836(92)90533-p

[30] Klenin K, V VA, Anshelevich VV, Dykhne AM, Frank-Kamenetskii MD (1991) Computer simulation of DNA supercoiling. Journal of Molecular Biology 217: 413–419. 1994032 10.1016/0022-2836(91)90745-r

[31] Levene S, Crothers D (1986) Topological distributions and the torsional rigidity of DNA: A Monte Carlo study of DNA circles. Journal of Molecular Biology 189: 73–83. 3783681 10.1016/0022-2836(86)90382-7

[32] Metropolis N, Rosenbluth AW, Rosenbluth MN, Teller AH, Teller E (1953) Equation of state calculations by fast computing machines. J Chem Phys 21: 1087–1092.

[33] Biton YY, Coleman BD (2010) Theory of the inuence of changes in salt concentration on the configuration of intrinsically curved, impenetrable, rod-like structures modeling DNA minicircles. International Journal of Non-Linear Mechanics 45: 735–755.

[34] Olson WK, Swigon D, Coleman BD (2004) Implications of the dependence of the elastic properties of DNA on nucleotide sequence. Phil Trans Roy Soc 362: 1403–1422. 10.1098/rsta.2004.1380 15306458

[35] El Hassan MA, Calladine CR (1995) The assessment of the geometry of dinucleotide steps in double-helical DNA: a new local calculation scheme. J Mol Biol 251: 648–664. 7666417 10.1006/jmbi.1995.0462

[36] Westcott TP, Tobias I, Olson WK (1997) Modeling self-contact forces in the elastic theory of DNA supercoiling. J Chem Phys 107: 3967–3980.

[37] Manning GS (1969) Limiting laws and counterion condensation in polyelectrolyte solutions: I. colligative properties. J Chem Phys 51: 924–933.

[38] Fenley MO, Manning GS, Olson WK (1990) Approach to the limit of counterion condensation. Biopolymers 30: 1191–1203. 2085657 10.1002/bip.360301305

[39] Vasilios I, Manousiouthakis V, Deem MW (1999) Strict detailed balance is unnecessary in Monte Carlo simulation. J Chem Phys 110: 2753–2756.

[40] Benham C, Mielke S (2005) DNA mechanics. Annu Rev Biomed Eng 7: 21–53. 16004565 10.1146/annurev.bioeng.6.062403.132016

[41] Gonzalez O, Maddocks JH (2001) Extracting parameters for base-pair level models of DNA from molecular dynamics simulations. Theoretical Chemistry Accounts: Theory, Computation, and Modeling (Theoretica Chimica Acta) 106: 76–82.

[42] White JH (1969) Self-linking and the Gauss integral in higher dimensions. Am J Math 91: 693–728.

[43] White JH (1989) An introduction to the geometry and topology of DNA structure. In: Waterman MS, editor, Mathematical methods for DNA Sequences. Boca Raton, Florida: CRC Press, pp. 225–253.

[44] Swigon D, Coleman BD, Tobias I (1998) The elastic rod model for DNA and its application to the tertiary structure of DNA minicircles in mononucleosomes. Biophys J 74: 2515–2530. 9591678 10.1016/S0006-3495(98)77960-3 PMC1299594

[45] Britton L, Olson W, Tobias I (2009) Two perspectives on the twist of DNA. J Chem Phys 131 245101 1–8. 10.1063/1.3273453 PMC2809498 20059113

[46] Ruben G, Roos T (1997) Conformation of Lac repressor tetramer in solution, bound and unbound to operator DNA. Microscopy Research and Technique 36: 400–416. 9140942 10.1002/(SICI)1097-0029(19970301)36:5<400::AID-JEMT10>3.0.CO;2-W

[47] Goeddel D, Yansura D, Caruthers M (1978) How Lac repressor recognizes Lac operator. Proceedings of the National Academy of Sciences 75: 3578–3582. 10.1073/pnas.75.8.3578 PMC392828 278973

[48] Sadler J, Sasmor H, Betz J (1983) A perfectly symmetric Lac operator binds the Lac repressor very tightly. Proceedings of the National Academy of Sciences 80: 6785–6789. 10.1073/pnas.80.22.6785 PMC390070 6316325

[49] Simons A, Tils D, von Wilcken-Bergmann B, Müller-Hill B (1984) Possible ideal Lac operator: Escherichia coli Lac operator-like sequences from eukaryotic genomes lack the central G X C pair. Proceedings of the National Academy of Sciences 81: 1624–1628. 10.1073/pnas.81.6.1624 PMC344970 6369330

[50] Müller J, Oehler S, Müller-Hill B (1996) Repression of Lac promoter as a function of distance, phase and quality of an auxiliary lac operator. Journal of Molecular Biology 257: 21–29. 8632456 10.1006/jmbi.1996.0143

[51] Priest D, Cui L, Kumar S, Dunlap D, Dodd I, et al (2014) Quantitation of the DNA tethering effect in long-range DNA looping in vivo and in vitro using the Lac and λ repressors. Proceedings of the National Academy of Sciences 111: 349–354. 10.1073/pnas.1317817111 PMC3890862 24344307

[52] Zurla C, Franzini A, Galli G, Dunlap D, Lewis DEA, et al (2006) Novel tethered particle motion analysis of ci protein-mediated DNA looping in the regulation of bacteriophage lambda. Journal of Physics: Condensed Matter 18: S225–S234.

[53] Finzi L, Dunlap D (2003) Single-molecule studies of DNA architectural changes induced by regulatory proteins. In: Adhya S, Garges S, editors, RNA Polymerases and Associated Factors, Part C, Academic Press, volume 370 of Methods in Enzymology. pp. 369–378. 10.1016/S0076-6879(03)70032-9 14712660

[54] Blumberg S, Gajraj A, Pennington MW, Meiners J (2005) Three-dimensional characterization of tethered microspheres by total internal reection uorescence microscopy. Biophysical Journal 89: 1272–1281. 15923224 10.1529/biophysj.105.061242 PMC1366611

[55] Han L, Lui BH, Blumberg S, Beausang JF, Nelson PC, et al. (2009) Calibration of tethered particle motion experiments. In: Benham CJ, Harvey S, Olson WK, Sumners DW, Swigon D, editors, Mathematics of DNA Structure, Function and Interactions, Springer New York, volume 150 of The IMA Volumes in Mathematics and its Applications. pp. 123–138.

[56] Milstein JN, Chen YF, Meiners JC (2011) Bead size effects on protein-mediated DNA looping in tethered-particle motion experiments. Biopolymers 95: 144–150. 20882535 10.1002/bip.21547

[57] Rhodes D, Klug A (1981) Sequence-dependent helical periodicity of DNA. Nature 292: 378–380. 6265794 10.1038/292378a0

[58] Tullius T, Dombroski B (1985) Iron(ii) edta used to measure the helical twist along any DNA molecule. Science 230: 679–681. 2996145 10.1126/science.2996145

[59] Bellomy G, Mossing M, Record M (1988) Physical properties of DNA in vivo as probed by the length dependence of the Lac operator looping process. Biochemistry 27: 3900–3906. 3046661 10.1021/bi00411a002

[60] Frank DE, Saecker RM, Bond JP, Capp MW, Tsodikov OV, et al (1997) Thermodynamics of the interactions of Lac repressor with variants of the symmetric Lac operator: effects of converting a consensus site to a non-speci_c site. Journal of molecular biology 267: 1186–1206. 9150406 10.1006/jmbi.1997.0920

[61] Segall DE, Nelson PC, Phillips R (2006) Volume-exclusion effects in tethered-particle experiments: Bead size matters. Phys Rev Lett 96 088306 1–4. 16606235 10.1103/PhysRevLett.96.088306 PMC3261840

